# Clinical analysis of the correlation between nutritional risk screening and prognosis of patients with chronic obstructive pulmonary disease

**DOI:** 10.12669/pjms.41.2.9566

**Published:** 2025-02

**Authors:** Qinghua Wu, Najuan Cui, BeiBei Wang

**Affiliations:** 1 Qinghua Wu Department of Respiratory, Beijing Hospital of integrated Traditional Chinese and Western Medicine Hospital of integrated Traditional Chinese and Western Medicine Beijing University of Chinese Medicine Beijing, 100039, China; 2 Najuan Cui Department of Respiratory, Beijing Hospital of integrated Traditional Chinese and Western Medicine Hospital of integrated Traditional Chinese and Western Medicine Beijing University of Chinese Medicine Beijing, 100039, China; 3 BeiBei Wang Department of Respiratory, Beijing Hospital of integrated Traditional Chinese and Western Medicine Hospital of integrated Traditional Chinese and Western Medicine Beijing University of Chinese Medicine Beijing, 100039, China

**Keywords:** Chronic Obstructive Pulmonary Disease, Prognosis, Nutritional Risk Screening, Influencing Factors, Correlation

## Abstract

**Objective:**

To explore the clinical correlation between nutritional risk screening and prognosis of patients with chronic obstructive pulmonary disease (COPD).

**Method:**

Clinical data of 80 patients with COPD admitted to Beijing Hospital of integrated Traditional Chinese and Western Medicine between August 2020 and August 2023 were retrospectively analyzed. Patients were divided into the Mortality group (n=17) and the survival group (n=63) according to the three years follow-up outcomes. The related data were compared between the two groups, the prognostic factors were analyzed, and the correlation between nutritional risk and prognostic risk factors was explored. The value of nutritional risk in prognosis prediction was analyzed by ROC curve.

**Result:**

Binary logistic regression analysis showed that complications (>2), NRS2002 score (nutritional risk), albumin (ALB), number of acute exacerbations of COPD (AECOPD) (≥2), pulmonary dysfunction (moderate to severe), and ventilator-associated pneumonia (VAP) after admission were prognostic risk factors (P<0.05). Complications, number of AECOPD, pulmonary dysfunction, and ALB were correlated with nutritional status (P<0.05). ROC curve analysis found that the AUC of NRS2002 score in prognosis prediction was 0.679(95%CI, 0.549-0.810), with a sensitivity of 88.2%, and a specificity of 47.6%, indicating the predictive value of this variable.

**Conclusion:**

Complications (>2), NRS2002 score (nutritional risk), ALB, number of AECOPD (≥2), pulmonary dysfunction (moderate to severe), and VAP after admission are prognostic risk factors. Nutritional status may be closely related to prognostic risk factors and might be used as a predictor for prognosis.

## INTRODUCTION

Chronic obstructive pulmonary disease (COPD) is a progressive lung disease characterized by restricted airflow, with clinical manifestations of dyspnea, cough, phlegm, and long-term smoking.^1^,^2^ Patients with COPD often experience decreased lung function, malnutrition, and weight loss during disease progression, which further impairs their quality of life (QoL) and prognosis.^3^ Nutritional risk screening refers to the comprehensive evaluation of the nutritional status of patients by using a series of assessment tools and methods to determine the presence of nutritional risk.^4^ The common nutritional risk screening tools currently used include Mini Nutritional Assessment (MNA) and Nutritional Risk Screening (NRS-2002). These tools are used to evaluate the nutritional status and risks by collecting relevant information such as weight changes, food intake, appetite, and oral health.^5^

Studies in recent years have found an increased nutritional risk in patients with COPD. Malnutrition is common in these patients, with an incidence of 30%-50%.^6^ The malnutrition in patients with COPD is possibly caused by factors such as dyspnea and inflammation and metabolic abnormalities induced the disease itself, which further exacerbates the conditions and impairs the lung function, immunity, and QoL of these patients. Studies have shown that the results of nutritional risk screening are related to the prognosis of patients with COPD, i.e., patients with a higher nutritional risk have a poorer prognosis.^7^ These results indicate that nutritional risk screening is of clinical significance in the prognosis prediction in patients with COPD.^8^ In the preset study, 80 patients with COPD were selected to explore the clinical correlation between nutritional risk screening and prognosis of patients with chronic obstructive pulmonary COPD.

## METHODS

This was a retrospective study. Eighty patients with COPD admitted to Beijing Hospital of integrated Traditional Chinese and Western Medicine between August 2020 and August 2023, and divided into the death group (n=17) and the survival group(n=63) according to the three years follow-up outcomes.

### Ethical Approval:

The study was approved by the Institutional Ethics Committee of Beijing Hospital of integrated Traditional Chinese and Western Medicine (No.: ZXYEC-KT-2022-03-P04; date: November 19, 2022).

### Inclusion criteria:

- Who met the relevant criteria in the Guidelines for the diagnosis and management of chronic obstructive pulmonary disease (revised version 2013)^9^
- With complete clinical data.
- With an age of ≥18 years old.

### Exclusion criteria:

- Severe dysfunction of important organs.
- Previous history of malignant tumors.
- Pulmonary diseases such as bronchiectasis, bronchial asthma and pulmonary fibrosis.
- Immune system diseases.
- Malignant tumors.
- Severe infections in other parts of the body.
- Metabolic diseases.
- Nutritional intervention and digestive system diseases.

The general data of the patients were collected, including sex, age, body mass index (BMI), smoking status, number of AECOPD, ALB level, arterial partial pressure of oxygen(PaO_2_), arterial partial pressure of carbon dioxide(PaCO_2_), oxygen saturation index (arterial partial pressure of oxygen/fractional inspired oxygen, PaO_2_/FiO_2_) on admission, use of noninvasive positive pressure ventilation(NIPPV) after admission, VAP or multiple organ dysfunction syndrome(MODS) after admission, and the NRS2002 score on admission.

Patients were screened for nutritional risk according to the Nutritional Risk Screening (NRS) Scale 2002 recommended by Chinese Medical Association Guidelines for Parenteral and Enteral Nutrition.^10^ This scale included three domains, i.e., disease severity, impaired nutritional status and age. The total score was the sum of the three scores, and a NRS score of ≥3 indicated the presence of nutritional risk.

### Pulmonary function assessment:

FEV_1_ >80% of predicted indicated mild pulmonary dysfunction; 50% < FEV1≤80% of predicted indicated moderate pulmonary dysfunction; and FEV1<50% of predicted or complicated with chronic respiratory failure indicated severe pulmonary dysfunction.^11^

The questionnaire was conducted face-to-face by investigators using standard, common and neutral language, and the nutritional status of patients was assessed on the day of admission. The questionnaire was completed anonymously. If the patients were unable to complete the questionnaire by themselves, the investigator truthfully completed the questionnaire on behalf of the patients in the form of questions and answers. The questionnaire was completed and collected on site, and numbered after invalid questionnaire was removed.

### Outcome Measures:

Relevant data were compared between the mortality group and the survival group, prognostic factors and correlation between nutritional risk and prognostic risk factors were analyzed. The value of nutritional risk in prognosis prediction was analyzed using ROC curves.

### Statistical Analysis:

SPSS22.0 was used for data analysis. Enumeration data were presented as n (%), and χ^2^ test was used for comparison between groups. Measurement data with normal distribution were presented as (`x±s), and independent sample t test was used for comparison between groups. Binary logistics regression was used to analyze the influencing factors, and ROC curve was used to analyze the diagnostic value. Differences with a p<0.05 were considered statistically significant.

## RESULTS

Univariate analysis showed no significant differences in age, sex, BMI, smoking, NIPPV, PaO_2_, PaO_2_/FiO_2_, and PaCO_2_ between the two groups (P>0.05 for all comparisons). However, there were statistically significant differences between the two groups in complications, NRS2002 score, ALB, number of AECOPD, pulmonary dysfunction, VAP after admission, and MODS after admission(P<0.05) (Table-I).

**Table-I:** Univariate analysis of the two groups.

Items	classification	The death group (n=17)	The survival group (n=63)	t/χ^2^	P
Age(years)		63.29±8.64	62.92±9.64	0.145	0.885
Sex	M	10(8.82)	42(66.67)	0.362	0.547
	F	7(41.18)	21(33.33)		
BMI (kg/m^2^)		21.94±2.83	22.43±2.6	0.668	0.506
Smoking	Yes	9(52.94)	19(30.16)	3.054	0.081
	No	8(47.06)	44(69.84)		
NIPPV	Yes	11(64.71)	36(57.14)	0.316	0.574
	No	6(35.29)	27(42.86)		
Complications	≤2	6(35.29)	41(65.08)	4.901	0.027
	>2	11(64.71)	22(34.92)		
PaO_2_(mmHg)		59.12±8.47	58.99±9.31	0.061	0.952
PaO_2_/FiO_2_(mmHg)		196.83±14.03	198.22±15.22	0.500	0.619
PaCO_2_(mmHg)		84.04±6.84	82.38±6.52	1.016	0.313
NRS2002 score	Normal nutrition	2(11.76)	30(47.62)	7.171	0.007
	Nutritional risk	15(88.24)	33(52.38)		
ALB(g/L)		32.19±3.72	40.83±5.23	6.442	<0.001
Number of AECOPD	0	4(23.53)	60(95.24)	35.463	<0.001
	1	9(52.94)	2(3.17)		
	≥2	4(23.53)	1(1.59)		
Pulmonary dysfunction	Mild	3(17.65)	19(30.16)	5.915	0.015
	Moderate	5(29.41)	32(50.79)		
	Severe	9(52.94)	12(19.05)		
VAP after admission	Yes	5(29.41)	6(9.52)	4.465	0.035
	No	12(70.59)	57(90.48)		
MODS after admission	Yes	4(23.53)	4(6.35)	4.390	0.036
	No	13(76.47)	59(93.65)		

Binary logistic regression analysis was conducted using prognosis as the dependent variable(0 = death, 1=survival), and complications, NRS2002 score, ALB, number of AECOPD, pulmonary dysfunction, VAP after admission, and MODS after admission as independent variables, and it was found that complications(>2), NRS2002 score(nutritional risk), ALB, number of AECOPD(≥2), pulmonary dysfunction(moderate to severe), and VAP after admission were risk factors for the prognosis(P<0.05) (Table-II and Table-III). Complications, number of AECOPD, pulmonary dysfunction, and ALB were correlated with nutritional status (P<0.05) (Table-IV).

**Table-II:** Variable assignments in logistic regression analysis of prognostic factors.

Variables	Variable Name	Assignment method
Prognosis	Y	Death=0, survival=1
Complications	X_1_	≤2 =0, >2 =1
NRS2002 score	X_2_	Normal nutrition=0, nutritional risk=1
ALB	X_3_	Continuous variable
Number of AECOPD	X_4_	0 time=0, 1 time=1, ≥2 times=2
Pulmonary dysfunction	X_5_	Mild=0, moderate=1, severe=2
VAP after admission	X_6_	Yes=0, No=1
MODS after admission	X_7_	Yes=0, No=1

**Table-III:** Logistic regression analysis of prognostic factors.

Variables	B	S.E.	Wald	df	*P*	OR	95% CI	

							Lower limit	Upper limit
Complications(>2)	1.229	0.572	4.611	1	0.032	3.417	1.113	10.487
NRS2002 score(nutritional risk)	1.920	0.794	5.846	1	0.016	6.818	1.438	32.319
ALB	0.341	0.085	16.218	1	<0.001	1.406	1.191	1.660
Number of AECOPD			25.731	2	<0.001			
1	-0.118	1.364	0.007	1	0.931	0.889	0.061	12.885
≥2	4.094	1.232	11.053	1	0.001	60.000	5.369	670.551
Pulmonary dysfunction			7.221	2	0.027			
Moderate	1.558	0.762	4.183	1	0.041	4.750	1.067	21.144
Severe	1.569	0.652	5.780	1	0.016	4.800	1.336	17.243
VAP after admission	1.376	0.684	4.049	1	0.044	3.958	1.036	15.120
MODS after admission	1.513	0.771	3.853	1	0.050	4.538	1.002	20.553

**Table-IV:** Correlation analysis between prognostic factors and nutritional status.

Items	Classification	Normal nutrition (32)	Nutritional risk (48)	t/χ^2^	P
Complications	≤2	14(43.75)	33(68.75)	4.952	0.026
	>2	18(56.25)	15(31.25)		
Number of AECOPD	0	23(71.88)	41(85.42)	5.838	0.016
	1	5(15.63)	6(12.50)		
	≥2	4(12.50)	1(2.08)		
Pulmonary dysfunction	Mild	6(18.75)	16(33.33)	6.758	0.009
	Moderate	14(43.75)	23(47.92)		
	Severe	12(37.50)	9(18.75)		
ALB(g/L)		41.35±4.88	37.41±6.30	2.980	0.004

**Fig.1:**
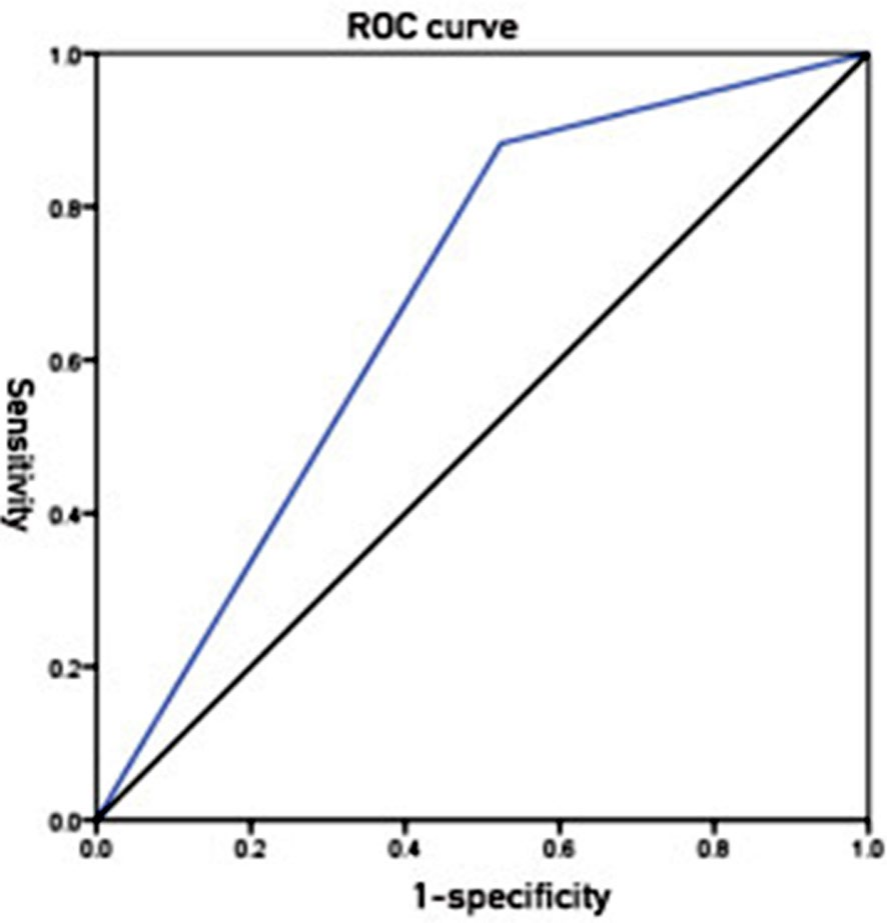
ROC curve of nutritional status (NRS2002 score) in prognosis prediction.

ROC curve analysis showed that the AUC of NRS2002 score in prognosis prediction was 0.679(95% CI, 0.549-0.810), with a sensitivity of 88.2%, and a specificity of 47.6%, indicating the predictive value of this variable (Fig.1).

## DISCUSSION

The results of the present study showed that complications (>2), NRS2002 score (nutritional risk), ALB, number of AECOPD (≥2), pulmonary dysfunction (moderate to severe), and VAP after admission were prognostic risk factors for COPD (P<0.05). The possible explanations included:

### Complications:

COPD is a complex disease with various complications, such as cardiovascular diseases and osteoporosis,^12^ and these comorbidities adversely affect the prognosis of patients;

### NRS2002 score:

The NRS2002 scale is a tool used to assess the nutritional status of patients and a lower score suggests a higher nutritional risk, which further induces malnutrition and related complications and consequently affects the outcomes of the patients;

### ALB:

Serum ALB is one of the indicators to evaluate the nutritional status and inflammatory response of patients. Lower ALB levels indicate malnutrition and inflammation, which adversely affect the prognosis of patients;

### Number of AECOPD:

Patients with AECOPD often require hospitalization, and frequent occurrence of AECOPD reflects the instability and worsening of the disease, which also adversely affects the prognosis of patients;

### Pulmonary dysfunction:

The severity of pulmonary dysfunction is closely related to the prognosis of patients with COPD.^13^ Moderate to severe pulmonary dysfunction indicates the significantly impaired pulmonary function, causing dyspnea and other complications;

### VAP after admission:

The development of VAP after admission is possibly caused by prolonged use of ventilator and often associated with ventilation-related infection and other complications and prolonged hospital stay, and adversely affects the prognosis of patients. The identification and assessment of these factors can help healthcare professionals to provide early intervention and improve the prognosis of patients.

The results of the present study showed that complications, number of AECOPD, pulmonary dysfunction and ALB were correlated with nutritional status (P<0.05), which was explained by the fact that complications, number of AECOPD, pulmonary dysfunction and ALB are affected by COPD, and these factors may work together on the nutritional metabolism, digestion and absorption functions of patients, leading to the deterioration of nutritional status. Patients with COPD are often complicated with persistent lung inflammation, causing metabolic disorders and impaired nutrient absorption and utilization in the body. Complications, number of AECOPD, pulmonary dysfunction and ALB are related to inflammation, and more severe inflammation is associated with more significant effect on nutritional status.^14^

Moreover, the circulatory and immune function in patients with COPD are often impaired, which further adversely affect their nutritional status.^15^ Complications, number of AECOPD, pulmonary dysfunction and ALB can serve as indicators for circulatory and immune function, and are correlated with nutritional status. The NRS2002 scale objectively reflects the nutritional status of patients by evaluating clinical parameters and nutrition-related factors.^16^ Patients with COPD often experience loss of appetite, and body weight loss, which directly affect their nutritional status. The NRS2002 scale can be used to comprehensively evaluate the nutritional status of patients and subsequently to predict their prognosis. Moreover, the NRS2002 scale provides a tool for patient-specific nutritional risk assessment, which is helpful for early identification of malnourished patients and appropriate interventions.

Measures such as reasonable nutritional support and dietary adjustment may promote the nutritional status of patients, reduce the development of complications, and finally improve the prognosis. The result of ROC curve analysis in the present study showed that the AUC of NRS2002 score in prognosis prediction was 0.679(95%CI 0.549-0.810), with a sensitivity of 88.2% and a specificity of 47.6%, indicating the favorable predictive effect of nutritional risk screening on the prognosis of patients with COPD and its predictive value. These results were consistent with the findings of studies by Wang Hui et, al.^17^

COPD is a progressive lung disease characterized by limited airflow, and often associated with smoking.^18^ Airflow restriction is primarily caused by thickened airway wall, excessive mucus secretion and airway contraction induced by chronic inflammation of airway and lung tissue.^19^ According to the World Health Organization (WHO), the morbidity and mortality of COPD remain high worldwide, and the latest data showed that the global prevalence of COPD is about 13.9%, i.e., about 130 million people suffer from COPD worldwide. China is one of the countries and regions with a high prevalence of COPD.

It is estimated that there are more than one hundred million patients with COPD in China, with an incidence of about 8%-13%.^20^ COPD is a serious danger to the health of patients. Progression of this disease is associated with worsened dyspnea, coughing, phlegm and other symptoms. Patients may be limited in their daily life due to dyspnea, and even unable to complete their regular daily activities.^21^ In addition, COPD can also lead to decreased pulmonary function, increasing the risk of AECOPD and respiratory failure.^22^ Meanwhile, patients with COPD are often complicated with other chronic diseases such as cardiovascular disease and osteoporosis, which further increase the severity of COPD. Malnutrition and dietary problems are very common in patients with COPD, and this is primarily explained by difficulty eating induced by dyspnea, gastrointestinal problems, COPD related inflammatory responses, and increased metabolism. Malnutrition, in turn, may further influence the prognosis of these patients. Therefore, nutritional risk screening is of great importance in the prognosis of patients with COPD. Early identification of patients with high nutritional risk and implementation of appropriated nutritional interventions can improve the prognosis and QoL of these patients.^23^

### Limitations:

However, this study also has some shortcomings, such as a small number of patients were included. In view of this, more patients should be included in future studies to further validate the findings of this study.

## CONCLUSIONS

In conclusion, complications (>2), NRS2002 score (nutritional risk), ALB, number of AECOPD (≥2), pulmonary dysfunction (moderate to severe), and VAP after admission are prognostic risk factors. Nutritional status is closely related to prognostic risk factors and may be used as a predictor for prognosis.

### Authors’ Contributions:

**QW:** Carried out the studies, data collection, drafted the manuscript, responsible and accountable for the accuracy or integrity of the work.

**NC:** Conceptualization and design, Critical review.

**BW:** Drafted the article, data interpretation.

All authors read and approved the final manuscript.
